# The Effectiveness of Art Activities and Peer Group Participation on Psychological Well-Being Among Older Adult Center Residents: Protocol for a Randomized Controlled Trial

**DOI:** 10.2196/85612

**Published:** 2026-05-29

**Authors:** Liping Pu, Praneed Songwathana

**Affiliations:** 1 Faculty of Nursing, Prince of Songkla University, Songkhla, Thailand; 2 School of Health Management, Suzhou Vocational Health College, Suzhou, China; 3 Research and Innovation Center for Well-being and Continuing Care, Department of Adult and Gerontology Nursing, Faculty of Nursing, Prince of Songkla University, 15 Kanjanawanich Road, Hatyai, Songkhla, 90110, Thailand, 66 815429170, 66 74286421

**Keywords:** art activity, peer group, psychological well-being, older adults, randomized controlled study

## Abstract

**Background:**

Transitioning to residential centers often induces significant psychological stress in older adults, adversely affecting their psychological well-being (PWB) and ability to age healthily. Chinese art activities and their combination with peer group participation have demonstrated efficacy in improving emotional well-being and fostering social connectivity among younger populations but have not been examined in older adult populations to date.

**Objective:**

This study aims to assess the efficacy of Chinese art activities or their combination with peer group participation in enhancing PWB among Chinese older adults living in a residential center.

**Methods:**

This multicenter randomized, assessor- and analyst-blinded trial uses a “max-min-con” design with 3 groups: control, Chinese art activities, and Chinese art activities plus peer group participation (1:1:1 allocation). A total of 90 participants aged 60 to 85 years and with a stay of 1 year or less will be recruited from 3 older adult centers (30 from each center) and assigned via minimized randomization to the groups. Interventions will consist of 3 sessions within a single week (Monday, Wednesday, and Friday). Baseline evaluations will be conducted before randomization to assess PWB, loneliness, happiness, relaxation, and salivary cortisol levels. Outcome evaluations will be conducted 30 minutes prior to and following each intervention session to assess immediate effects. A follow-up evaluation is planned 1 week after the final intervention to measure sustained outcomes.

**Results:**

The study was funded in September 2024. Participant recruitment commenced on May 9, 2025, and ended on August 20, 2025, with the recruitment of 90 participants. Data collection ended on October 31, 2025, and data analysis is expected to conclude in April 2026, with the anticipated publication of results in 2026.

**Conclusions:**

This study is the first randomized controlled trial to evaluate the effects of Chinese art activities, either alone or combined with peer group participation, on enhancing PWB in older people and the efficacy of salivary cortisol as a biomarker for the assessment of PWB. The findings will provide empirical evidence to assist policymakers and older adult centers in selecting appropriate interventions, with the long-term potential to improve psychological health in older adults.

## Introduction

### Background

The global older adult population is growing rapidly, posing significant social and economic future challenges. In China, the 1-child policy has exacerbated older adult care difficulties, increasing reliance on institutional care. However, transitioning to older adult centers often induces stress [1-3]; reduces psychological well-being (PWB); and can cause physical symptoms such as insomnia and appetite loss [4], which lead to worsened health [5]. In China, which has historically relied on home-based care, relocation to older adult centers can heighten feelings of neglect and embarrassment [3], further compromising PWB.

Maladaptation in older adults is more likely to occur in the first year of entry to older adult centers as they face the loss of familiar surroundings and must adapt to a new environment [6]. These phenomena may be of heightened concern in Chinese culture, where traditional values of filial piety emphasize children caring for their older parents. The admission to older adult centers for older adults may lead to feelings of abandonment and shame. Consequently, a decline in PWB is more common among new residents, especially in the Chinese context [7,8]. Therefore, researching ways to improve the PWB of new residents is highly meaningful.

Recent studies have explored behavioral interventions such as happiness therapy [9,10], life review therapy [11,12], and cognitive behavioral therapy [13-15] to improve PWB. A limitation of these interventions is that they are often perceived as uninteresting, which may promote disengagement among older adults [16]. Additionally, many interventions primarily target younger adult populations, further limiting their applicability [17-20]. Another significant drawback is that these studies often rely solely on subjective data, lacking support from objective measurements, which may affect the validity and reliability of the findings [21-24]. Therefore, it is essential to develop practical, effective, and easily accepted interventions specifically designed to improve PWB among older adults while incorporating both subjective and objective data to ensure more robust and comprehensive results.

Art activities and group-based interventions are widely recognized as effective methods for enhancing PWB [25,26]. Some studies [27-30] have incorporated group discussions or supplementary activities into art-based interventions to improve outcomes. While these combined interventions have potentially shown some effectiveness, this benefit comes with the drawback of a longer duration, which may inadvertently introduce negative effects such as participant fatigue or reduced adherence [31]. Therefore, it is critical to examine whether art activities alone or in combination with group discussions are more effective in boosting PWB among new residents in Chinese older adult care centers.

### Conceptual Framework

**Figure 1.**
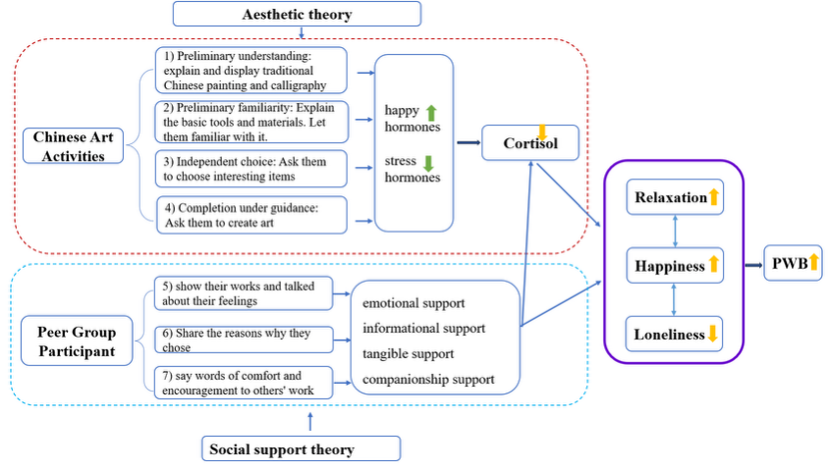
Conceptual framework of the study. PWB: psychological well-being.

The study is based on aesthetic theory and social support theory. Aesthetic theory underscores the significance of aesthetic experience in enhancing well-being via emotional and sensory reactions to art [32,33]. Interaction with beauty is correlated with favorable emotional states, which neuroscience links to the release of neurotransmitters such as endorphins and serotonin as well as a decrease in stress-related indicators, including cortisol [34-37]. Social support theory emphasizes that interpersonal interactions mitigate stress and improve well-being by providing emotional, informational, and companionship support [38-42]. Aesthetic theory corresponds to the creative process of Chinese painting and calligraphy in the Chinese art activity component of this intervention, mainly supporting the mechanisms of changes in emotional states and self-esteem levels. Social support theory corresponds to the peer group participation component, mainly guiding the mechanism of changes in perceived social support and loneliness. The concept structure of this study is shown in Figure 1.

This study is an exploratory trial. PWB, happiness, relaxation, loneliness, and salivary cortisol levels were selected as the dependent variables. Happiness, relaxation, and loneliness are all operationally defined as an immediate, subjective, and transient affective state characterized by momentary feelings [43,44]. Usually, PWB requires intervention for a longer period to manifest changes. However, as an exploratory trial, we still included it as an exploratory outcome measure to provide preliminary data for future research.

### Objectives

The objectives of the trial are to establish whether (1) new residents in Chinese older adult centers have higher PWB scores, happiness, and relaxation, as well as lower levels of loneliness and salivary cortisol, after engaging in Chinese art activities; (2) new residents in Chinese older adult centers have higher PWB scores, happiness, and relaxation, as well as lower levels of loneliness and salivary cortisol, after engaging in Chinese art activities and peer group participation; (3) new residents in Chinese older adult centers engaging in a Chinese art activity and peer group participation intervention have higher PWB scores, happiness, and relaxation, as well as lower levels of loneliness and salivary cortisol, than those who only engage in Chinese art activities; and (4) salivary cortisol can serve as a biomarker reflecting changes in PWB and relaxation.

On the basis of aesthetic theory and social support theory, we propose the following hypotheses: (1) for objectives 1 and 2, we hypothesize that both Chinese art activities and Chinese art activities with peer group participation will promote PWB, happiness, and relaxation and alleviate loneliness; (2) for objective 3, we hypothesize that peer group participation will have a stronger effect than Chinese art activities; and (3) for objective 4, we hypothesize that salivary cortisol will be a biological indicator of PWB and relaxation.

## Methods

### Pilot Study

Prior to the trial, we conducted a pilot study with 20 older adults (10 per group) recruited from an older adult center in Suzhou that is not affiliated with the authors’ institutions in December 2024. The objectives were to assess feasibility and acceptability and refine intervention protocols. Recruitment achieved 100% of the target within 2 weeks. A total of 95% (19/20) of the participants attended all 3 sessions, with 1 dropout due to a spontaneous family trip. All assessment instruments were completed without missing data, and the saliva collection procedure was well tolerated (no refusals). Participants reported that the activities were enjoyable and easy to follow.

On the basis of the findings of the pilot study, the following improvements were made to the main experiment. The intervention time from morning to afternoon was adjusted based on the suggestions and preferences of the participants. Due to the widespread feedback from participants (10/15, 66.7%) that the Chinese calligraphy activity was not completed in time, the duration of this stage was extended from 10 minutes to 15 minutes. We further simplified the painting template to address the difficulty of Chinese painting raised by participants. Guidance was also added to the peer volunteer training on managing quiet participants.

The revised protocol obtained official approval before its implementation in the main study.

### Trial Design

This multicenter randomized, assessor-blinded trial is structured according to the “max-min-con” principle [45,46]: maximizing experimental variance by ensuring that art activities and art activities with peer group participation are delivered consistently and distinctly from the control condition; minimizing error variance by using standardized protocols for art activities and art activities with peer group participation, trained research assistants (RAs), and reliable measurement instruments; and controlling extraneous variance through minimized randomization and blinding of outcome assessors.

**Figure 2.**
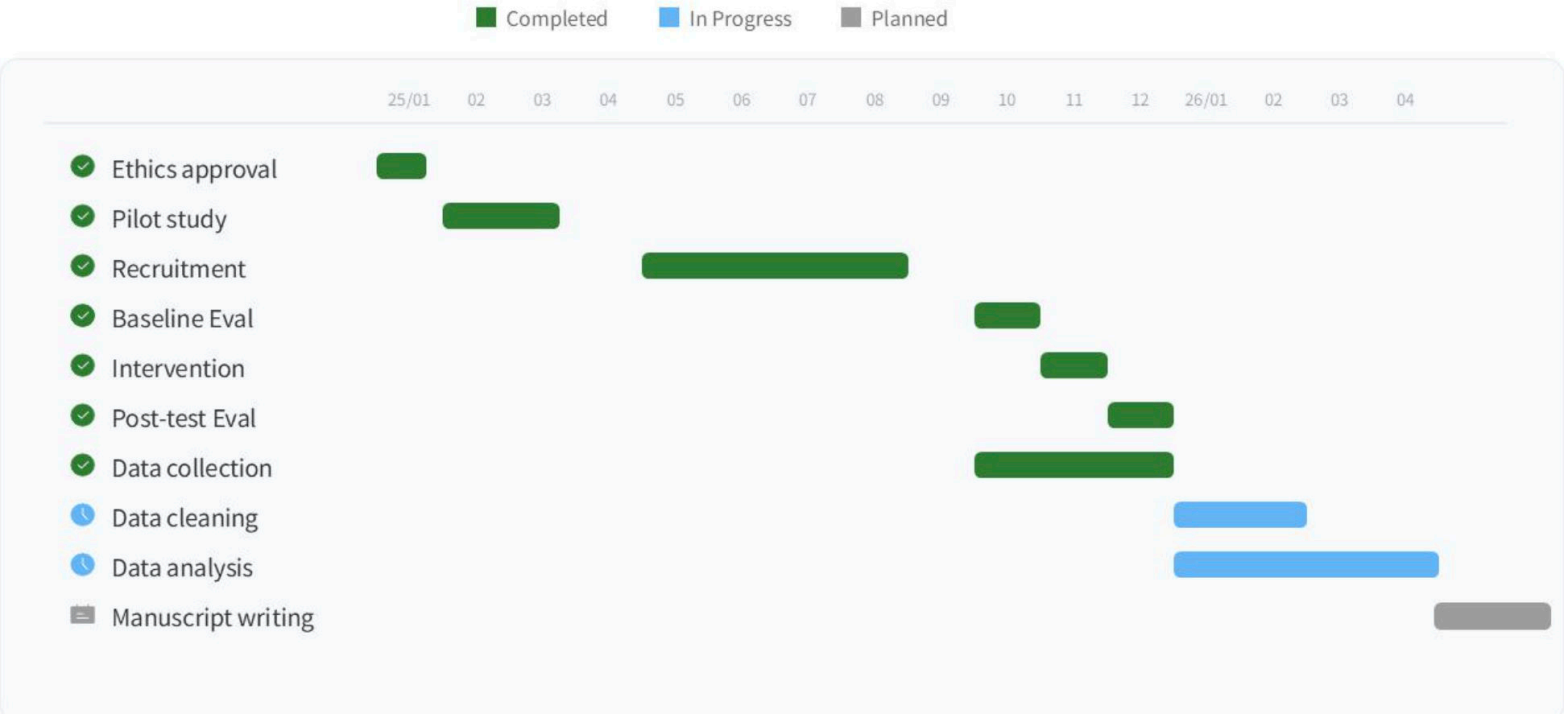
The implementation phase and data collection procedures.

This study has 3 intervention groups: group A (Chinese art activities), group B (Chinese art activities+peer group participation), and group C (regular care), with participants randomized at a 1:1:1 allocation ratio. The study will be conducted consecutively at 3 older adult centers. These 3 centers all belong to Suzhou Health and Elderly Care Industry Development Group Co, Ltd. After on-site inspection, it was determined that the inclusion criteria (affiliation, bed capacity, facilities, management support, and accessibility) were met to ensure feasibility and reasonable representativeness. Each center is treated as a randomization unit. Figure 2 delineates the implementation phase and the data collection procedures in the study. The intervention takes place over 3 sessions in a single week (Monday, Wednesday, and Friday). Baseline evaluations of PWB, loneliness, happiness, relaxation, and salivary cortisol levels are conducted before randomization.

**Figure 3.**
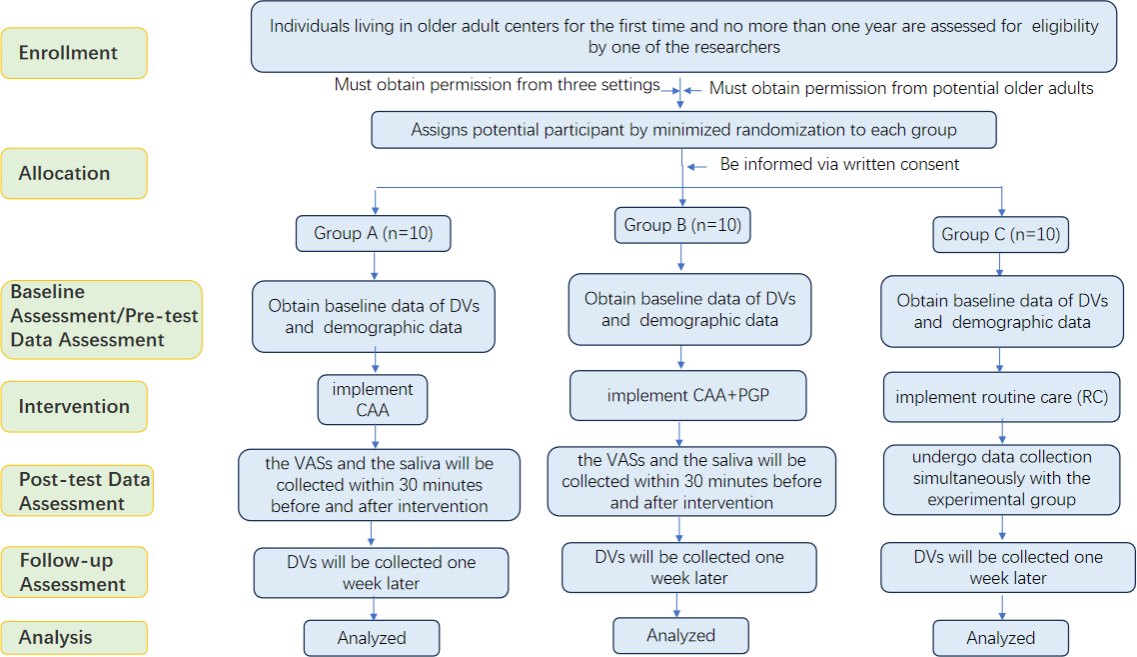
CONSORT (Consolidated Standards of Reporting Trials) flow diagram. CAA: Chinese art activities; DV: dependent variable; PGP: peer group participation; RC: routine care; VAS: visual analog scale.

Outcome measures are gathered 30 minutes prior to and following each intervention session to assess immediate effects. A concluding follow-up assessment takes place 1 week after the final session to examine enduring outcomes. Figure 3 illustrates the research design within a single environment. The study protocol (version 1.0 dated March 2, 2026) complies with the SPIRIT (Standard Protocol Items: Recommendations for Interventional Trials) checklist (Table 1).

**Table 1.** SPIRIT (Standard Protocol Items: Recommendations for Interventional Trials) table with the schedule of enrollment, interventions, and assessments.

	Study period					
	Enrollment (T–1)	Allocation (T0)	Intervention session—Monday (T1)	Intervention session—Wednesday (T2)	Intervention session—Friday (T3)	Intervention session—following Monday (T4)
Enrollment						
Eligibility screening	✓					
Information sheet	✓					
General informed consent	✓					
Group-specific informed consent		✓				
Allocation		✓				
Interventions						
Usual care			✓	✓	✓	
Usual care+CAA^a^			✓	✓	✓	
Usual care+CAA+PGP^b^			✓	✓	✓	
Assessments						
PWB^c^ score	✓					✓
Loneliness score—VAS^d^	✓		✓^e^	✓^e^	✓^e^	✓
Happiness score—VAS			✓^e^	✓^e^	✓^e^	✓
Relaxation score—VAS			✓^e^	✓^e^	✓^e^	✓
Salivary cortisol level			✓^e^	✓^e^	✓^e^	✓

a CAA: Chinese art activities.

b PGP: peer group participation.

c PWB: psychological well-being.

d VAS: visual analog scale.

e This includes pretest and posttest assessment.

### Participants

Eligible participants are aged 60 to 85 years, residing in an older adult center with a duration of stay not exceeding 1 year [47] and exhibiting no cognitive function, defined as a Mini-Mental State Examination (MMSE) score above 24 [48]. Additionally, participants are required to have adequate skills for basic reading and writing [49] and provide voluntary informed consent. Exclusion criteria encompass severe medical conditions such as heart failure, asthma, cerebrovascular disease, or advanced malignancies, as well as planned or actual discharge from the facility during the study period.

### Sample Size

The sample size of this randomized controlled trial (RCT) was calculated based on a previous study. The effect size used the formula [50].

In this formula, *d* is the effect size, μ1 is the mean of the intervention group, and μ2 is the mean of the control group.


$$\sigma =\sqrt{\text{SD}1^{2}+\text{SD}2^{2}}/2$$

Where SD₁ and SD₂ are the standard deviations of the control group and the intervention group, respectively. The term σ represents the pooled SD, calculated under the assumption that the two groups have equal sample sizes. This pooled SD is used to compute Cohen *d* effect size. The sample size calculation is based on the loneliness effect size (*d*=0.92) from the study by Aydın and Kutlu [51], which we selected as the most conservative among the 3 available effect sizes (the other 2 being happiness [*d*=2.66] and relaxation [*d*=2.32]) [52]. Using a power chart [53], the sample size was determined based on an effect size of 0.92, a power of 0.80, and an α level of .05. The analysis indicated that a minimum of 25 participants per group would be required. This study will recruit 90 individuals to account for a potential dropout rate of 20%. The trial is scheduled at 3 independent institutions, each having 3 groups with 10 participants per group.

### Participant Enrollment

Upon receiving formal authorization from the management of the older adult centers, the nursing director acts as the principal gatekeeper, enabling researcher access to the facility and aiding in the recruitment of prospective participants. Eligible participants are determined by examining health records and MMSE scores in accordance with the inclusion criteria. Individuals who satisfy the requirements are invited to participate and provided with a written information sheet detailing the study’s aim, procedures, and advantages and their right to leave without repercussions. Written informed consent is requested prior to enrollment. For group allocation, participants obtain a group-specific information sheet and sign a corresponding consent form.

### Participant Allocation

The allocation sequence is produced dynamically by an independent statistician throughout the recruiting phase. Each research site serves as a randomization unit. Using the Minimization Random Program software (version 2.01) [54,55], 30 eligible participants are randomly divided into 2 experimental groups and 1 control group in a 1:1:1 ratio, with 10 participants in each group. The confounding variables are age and number of visits per month. A study found that happiness increases with age before the age of 75 years, but for those over 75 years, the probability of happiness begins to decrease. Therefore, participants will be categorized into age groups of 60 to 75 years and over 75 years [56]. The other confounding variable is the number of visits per month. Older people living in institutions tend to meet with family and friends more frequently, often having more positive and less negative feelings [57]. Hence, participants were classified as having visits 1 to 2 times per month or 3 or more times per month. The categories of confounding variables are shown in Table 2.

The researchers will recruit 30 participants from each institution, resulting in a total of 90 older adults (aged 60‐85 years; individuals with ≤1 year of residence) referred by health care providers. In each institution, the 30 participants are allocated into 3 groups—A, B, and C—comprising 10 participants each. Group A is administered Chinese art activities, group B is administered Chinese art activities and peer group participation, and group C receives routine care.

**Table 2.** Categories of confounding variables in the minimized randomization program.

Confounding variable	Category 1	Category 2
Age (y)	60-75	>75
Monthly visit frequency	1-2 times	≥3 times

### Blinding

A double-blind design is impractical due to the characteristics of the art-based intervention; however, outcome assessors and data analysts will remain blinded to guarantee an objective review. RAs responsible for pre- and postintervention data collection remain uninformed of group assignments and will not engage in the delivery of the intervention. An unblinded RA manages randomization and intervention delivery but is excluded from data collection tasks. Data analysts receive solely anonymized participant codes devoid of any information regarding group allocation. All blinded staff are not able to obtain information on group assignments.

### Intervention

#### Chinese Art Activities

A systematic, structured protocol guide is followed for the implementation of the Chinese art activity intervention at all the research centers [58-60]. The program begins with a 10-minute instructional introduction presented through a preprepared Microsoft PowerPoint presentation exhibited on a wide screen. These sessions focus on the historical development, philosophical underpinnings, and essential brush-and-ink techniques of traditional Chinese landscape painting and calligraphy. Example artworks are displayed on the screen throughout the relevant instructional segments to demonstrate aesthetic principles and improve participants’ visual literacy. A 3-minute instructional movie is subsequently shown. An iPad (Apple Inc) is supplied and positioned on the desks of participants who indicate difficulties with reading the screen clearly. The movie systematically illustrates the creation of a landscape (meticulously chosen by teacher or professor) scene using novice-friendly brush techniques and color modification methods. Afterward, participants have 25 minutes to replicate or modify (brush and color) samples on rice paper using traditional tools such as wolf brushes, pine smoke ink, and mineral pigments. Personalized design through color changes or the addition of natural elements such as birds and flowers is intentionally promoted to enhance individual creative expression. The next calligraphy activity is arranging for these participants to write a short lyrical poem or motto with a consistent theme based on their own paintings. During this phase, participants are assigned individual desks and chairs and independently engage in Chinese painting and calligraphy creation. Therefore, it is difficult for participants to communicate with each other.

This intervention is implemented by one of our RAs. He is a university professor and has studied Chinese painting and calligraphy for 5 years. He also has experience in organizing group activities. Standardized training is required for him on Chinese art activity intervention procedures, protocols, and security precautions. A detailed summary of the interventional strategy for Chinese art activities is provided in Textbox 1.

Textbox 1. **Details of the Chinese art activity program.**

**Activity**

- Traditional Chinese landscape painting and calligraphy

**Instruments used**

- Pens, ink, paper, inkstones, and pigments

**Procedure and duration**

- Preliminary understanding and familiarization (10 minutes)
- Creation of traditional Chinese landscape paintings (25 minutes)
- Chinese calligraphy creation (15 minutes)

#### Chinese Art Activities Combined With Peer Group Participation

The Chinese art activities with peer group participation intervention, supported by previous studies [51,61], consists of an 80-minute integrated session led by a single researcher (RA). On the basis of established recommendations that at least 4 participants are necessary for effective group interventions [62], the 10 participants in this arm are randomly divided into 2 groups of 5, each facilitated by 1 trained peer volunteer, resulting in 6 individuals per group including the peer volunteer.

Peer volunteers are recruited through self- and staff recommendation. The inclusion criteria are being aged over 60 years, having lived in the center for more than 6 months, having normal cognition, moving freely, having good communication skills, and being enthusiastic to participate. They are responsible for organizing and guiding participants to actively express themselves and encourage each other during the peer group participation phase. One week before the intervention begins, training on research objectives and intervention procedures is conducted through meetings. The training also includes researchers demonstrating communication skills such as organizing peer evaluations, encouraging expression, and strengthening practical abilities through simulated exercises and on-site guidance. The training simultaneously evaluates the competence of volunteers, screening qualified candidates to participate in subsequent interventions. This study will not provide financial compensation to peer volunteers. As an alternative, the research team will give them flowers.

The intervention begins with Chinese art activities that follow the established procedures. After the Chinese art activities are completed, peer group participation is immediately carried out. Peer volunteers organize members to showcase their works. They invite each member to elaborate on the significance of their work while promoting mutual appreciation among group members. Subsequently, participants are encouraged to exchange their works as gifts accompanied by handshakes, smiles, or nods. Finally, peer volunteers conclude the program by sharing personal stories highlighting adaptation to life at the older adult center. Textbox 2 contains information pertaining to the Chinese art activities and peer group participation interventional procedures.

The intervention is implemented 3 times within a week following the structure described in Textbox 2 for each implementation. However, the content of each art activity slightly changes, involving drawings of watermelons, peaches, and lychees to maintain participation.

This study is about entertainment activities, and there are no adverse side effects. The research will last for 1 week, so no midterm analysis will be conducted. The entertainment activity arranged by the center does not involve the termination of the research. Hence, there is no trial monitoring either.

Textbox 2. **Details of the Chinese art activity and peer group participation program.**

**Activities**

- Traditional Chinese landscape painting and calligraphy and peer group participation

**Instruments used**

- Pens, ink, paper, inkstones, and pigments

**Procedure and duration**

- Preliminary understanding and familiarization (10 minutes)
- Creation of traditional Chinese landscape painting (25 minutes)
- Chinese calligraphy creation (15 minutes)
- Display and appreciation of artworks (5 minutes)
- Discussion of ongoing individual art activities (25 minutes)
- End of the intervention

#### Routine Care

As all 3 older adult centers operate under the same management, they follow the same schedules. These include daily activities such as basic limb exercises during specific activity hours from 8:30 AM to 10:30 AM and 2 PM to 4 PM. The Chinese art activities and Chinese art activities with peer group participation interventions are planned from 2 PM to 4 PM, whereas the control group will adhere to the original activity schedule of the older adult centers. All other everyday management and activities for the 3 groups will continue as normal.

### Outcomes

This research uses a repeated-measure design. In addition to the measurements obtained concurrently with the PWB assessment at baseline and 1 week after the intervention, further pre- and postintervention evaluations are performed for the happiness, relaxation, and loneliness scores and salivary cortisol levels. The schedule of evaluations is outlined in Table 1.

Demographic variables include age, gender, marital status, number of children, educational level, number of chronic diseases, type of residence (private or shared room), length of stay in the older adult center, frequency of monthly visits, and MMSE score.

The PWB score is assessed using the 18-item Chinese version of the PWB scale by Ryff [63]. This scale consists of 18 questions with 3 questions per dimension, covering a total of 6 dimensions: self-acceptance, positive relations with others, autonomy, environmental mastery, purpose in life, and personal growth.

**Figure 4.**
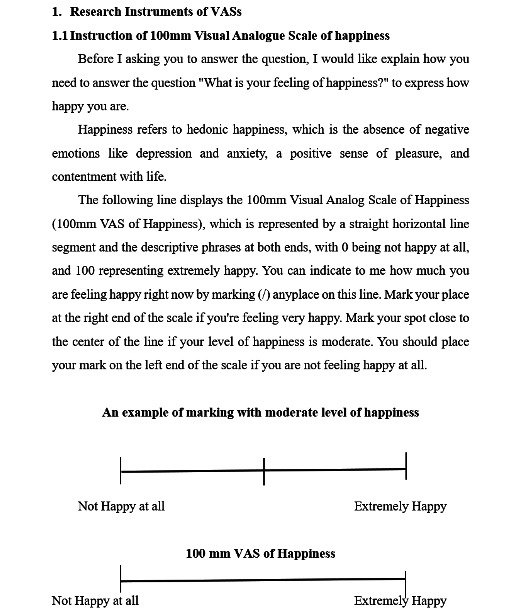
Visual analog scale (VAS) for happiness.

The happiness, relaxation, and loneliness scores are measured using a specific 10-cm visual analog scale (VAS) [64-67]. After a decision is made, the recorded value is measured in millimeters (from left to right) using a ruler to assign a numerical value to the subjective evaluation, with possible scores ranging from 0 to 10. An example of the VAS is shown in Figure 4.

The VAS is a widely recognized and proven effective evaluation tool for subjective experience measurement in the older adult population [64,68-72]. However, studies have also shown that patients with cognitive decline, decreased hand flexibility, and impaired vision may find it difficult to complete, leading to unreliable results [71,73,74]. Therefore, the inclusion criteria stipulated requirements for MMSE scores higher than 24 and the ability to independently read and write. At the same time, the scale uses clearly marked and high-contrast black lines to ensure visual clarity, and during the first use, researchers will explain and demonstrate how to complete the scale to ensure that effective results are obtained from the participants.

Saliva will be collected 30 minutes before and following the intervention. We will use a saliva collecting tube. After collection, the saliva samples will be put on wet ice right away, brought to a laboratory within 3 hours, and frozen at −80 °C. Following the collection of all samples, the laboratory’s qualified researcher will be asked to conduct the salivary cortisol measurements on all the collected samples. The Jiangsu Provincial Department of Science and Technology has accredited the laboratory, and it has significant proficiency in measuring salivary cortisol levels. We will take the following measures to improve the effectiveness of cortisol measurement: (1) participants are instructed to avoid drinking alcohol for 12 hours, avoid eating for 1 hour, and avoid brushing their teeth for 10 minutes before collection; (2) all saliva samples are collected between 2 PM and 4 PM; and (3) samples contaminated with blood are excluded. The reference range for salivary cortisol is 2.09 to 22.63 nmol/L [75].

### Statistical Methods

This study will use computer-based data analysis with the following statistical procedures.

After data collection is completed, the RA will review and remove any missing data. Before inputting the results into the computer to create a dataset, all data need to be checked, and errors need to be corrected. Descriptive statistics will be used to analyze and describe the demographic data of the participants.

In this study, the dependent variables are PWB, happiness, loneliness, relaxation, and salivary cortisol levels, and the independent variables are interventions (Chinese art activities, Chinese art activities+peer group participation, and routine care). Descriptive statistics will be used to check for missing values, out-of-range data, and data accuracy. Histograms, skewness, and kurtosis will be used to test the normality of the dependent variables in each group. If the data follow a normal distribution, the mean and SD will be used for analysis. If they are not normally distributed, the median and IQR will be used to represent them.

For the testing of objectives 1 and 2, a dual test of intra- and intergroup comparisons will be used. If the data are normally distributed, 2-tailed paired-sample *t* tests will be used for intragroup comparison of the Chinese art activities group or the Chinese art activities and peer group participation group. This will test the intervention effects of the Chinese art activities or Chinese art activities with peer group participation interventions on happiness, relaxation, loneliness, and salivary cortisol levels before and after the intervention (3 times). We will use independent-sample *t* tests to compare the effects of the Chinese art activities or Chinese art activities with peer group participation interventions on happiness, relaxation, loneliness, and salivary cortisol levels with the control group. For the testing of objective 3, an independent-sample *t* test will be used to compare the effects of the Chinese art activities with peer group participation and the Chinese art activities interventions on happiness, relaxation, loneliness, and salivary cortisol levels. For the testing of objective 4, salivary cortisol levels will be the main independent variable, and changes in PWB or relaxation will be divided into 3 quartiles as dependent variables. Ordered logistic regression analysis will be used to explore the association between salivary cortisol and these 2 variables. There will be no additional analyses.

Due to the harmless and short-term nature of this study, there will be very little missing data. Preliminary analysis will be conducted based on the complete case. We will use mean imputation for any missing data within a scale. Following the intention-to-treat principle, participants with intervention data will be included in the analysis.

### Ethical Considerations

This study protocol has been reviewed and approved by the institutional review board of Prince of Songkla University (approval 2024-St-Nur 048). The trial is registered at ClinicalTrials.gov (NCT06841133). Any modifications to the protocol will be promptly submitted to the institutional review board for review and approval.

Before participation, all eligible individuals receive detailed information regarding the study objectives, procedures, potential risks, and benefits. Written informed consent is obtained from each participant after a full explanation of the study requirements has been provided. For participants with visual or literacy challenges, the consent form is read aloud verbatim by a trained RA, and their written signature is obtained. To ensure confidentiality, all collected data are deidentified using unique participant codes. Electronic data are stored on encrypted, password-protected servers, and physical documents are kept in locked cabinets accessible only to authorized investigators. Saliva samples are processed for cortisol analysis immediately after collection and destroyed thereafter to prevent unauthorized use or retention. All research personnel have completed training in ethical data handling and participant privacy protection according to international standards [76].

In this study, the experimental group will receive supplemental entertainment activities as interventions alongside routine activities, whereas the control group will receive only routine activities. After the trial, the control group will be offered the same interventions if interested without follow-up requirements.

## Results

The study was funded in September 2024. The first participant was enrolled on May 9, 2025. A total of 90 participants have been recruited as of August 20, 2025. Data collection ended on October 31, 2025, and data analysis is expected to conclude in April 2026, with the anticipated publication of results in 2026.

## Discussion

### Anticipated Findings

To our knowledge, this is the first exploratory study on the improvement effect of traditional Chinese art activities on PWB among individuals who are new residents of older adult centers. On the basis of our pilot study demonstrating the feasibility and potential of Chinese art activities and Chinese art activities with peer group participation, this multicenter RCT aims to provide preliminary evidence on the effectiveness of these interventions in institutional environments. By comparing the outcomes of both interventions, we expect to determine the best program that is suitable for individuals who are new residents of older adult centers. At the same time, we speculate that combining subjective scales with the objective indicator of salivary cortisol can effectively verify the positive effects of Chinese art activities and Chinese art activities with peer group participation and salivary cortisol will significantly correlate with the subjective results of PWB, which will further confirm the physiological basis for the effects of these interventions.

### Comparison to Prior Work

To promote PWB, previous studies have pointed out that more high-quality art activity experiments are needed [77,78]. Our study will be a rigorously designed RCT conducted simultaneously in 3 older adult centers. In China, research on PWB interventions for older people in institutional care is still in the exploratory stage, with problems such as lack of intervention studies, excessive use of intervention methods that are not adapted to Chinese culture, and use of only subjective indicators for outcome evaluation [79,80]. This study incorporates Chinese painting and calligraphy, 2 traditional Chinese cultural elements, into the design of the Chinese art activities and Chinese art activities with peer group participation interventions, addressing the cultural adaptability issue of previous intervention methods. The study includes salivary cortisol as an objective variable that, combined with subjective scales, will enhance the reliability of the research results. The multicenter research design helps expand the sample size and enhances the scientific and rigorous nature of the results.

Most existing art activity interventions are combined with various group interactive activities [11,12], which increases the duration of the entire intervention process, increases the physical function requirements for older adults, and may reduce the effectiveness of the intervention. This study will compare the effects of simple art interventions combined with group activities and screen out intervention modalities that are more time-consuming and less suitable for older adults [31,81], providing evidence-based support for determining intervention plans that are suitable for the physical and mental state of new residents in older adult centers.

### Limitations

The intervention in this study will be implemented 3 times with a duration of only 1 week. Although results will be measured 1 week later, the total study time does not exceed 1 month. This may not be a major limitation for exploration research, but it is far from enough to expect long-term results. In addition, a short-term intervention is not conducive to the establishment of peer relationships. This may lead to the correctness of the relevant research results, which needs to be taken into account when interpreting the research results at the later stage in an actual RCT. It is recommended that future studies extend the duration to at least 3 months.

In our data collection process, VASs will be used for happiness, loneliness, and relaxation before and after the interventions. This repeated measurement in a short period can easily lead to practice effects and response shifts, resulting in measurement bias and a decrease in experimental validity. Therefore, when interpreting the data in this study, it is necessary to fully consider this limitation.

### Future Directions

Future research could extend the study period to more than 3 months to track the stability of intervention effects. A qualitative research design could also be added to analyze results from multiple dimensions. In addition, it is recommended to increase the number of physiological indicators, such as pulse and blood pressure, to enhance the objectivity of the research results.

### Dissemination Plan

We are preparing to submit our research findings to high-quality journals in fields such as geriatrics and nursing (eg, *JMIR Nursing*). The findings will also be shared at relevant academic conferences through oral presentations, poster displays, and other formats. We also plan to develop practical guidelines for the Chinese art activities and Chinese art activities with peer group participation interventions based on research evidence and promote them to older adult care institutions nationwide.

### Conclusions

This study will provide evidence-based support for incorporating Eastern wisdom into nursing practice. The development and implementation of culturally adapted art activity interventions for new residents of Chinese older adult centers has the potential to yield significant insights into the delivery and policies of aged care. The findings could inform the creation of practical guidelines for enhancing PWB in older adult centers by demonstrating the efficacy of culturally customized art-based interventions. The study emphasizes the significance of incorporating cultural traditions into contemporary care systems, presenting a scalable approach that may be applied to other aged care cultural contexts.

## Abbreviations

MMSE: Mini-Mental State Examination
PWB: psychological well-being
RA: research assistant
RCT: randomized controlled trial
SPIRIT: Standard Protocol Items: Recommendations for Interventional Trials
VAS: visual analog scale
